# Supplementary material for: An open-source framework for large-scale, flexible evaluation of biomedical text mining systems
Source: J Biomed Discov Collab. 2008 Jan 29;3:1. doi: 10.1186/1747-5333-3-1 (PMC2276192; doi:10.1186/1747-5333-3-1)
Supplement: Additional file 1 — Evaluation platform source code. The additional file contains the software evaluation framework discussed in this paper. The most current release can be downloaded from SourceForge.net [1]. See the accompanying README file for instructions on installation and use. Also included in the distribution is a collection of UIMA wrappers for some commonly used BioNLP tools and annotated corpora. [file 1747-5333-3-1-S1.zip › bionlp_uima_repository-1.0.1/docs/api/src-html/edu/uchsc/ccp/uima/annotation/syntactic/CCPTypedDependency.html]

```
001    
002    
003    /* First created by JCasGen Tue Aug 28 16:44:10 MDT 2007 */
004    package edu.uchsc.ccp.uima.annotation.syntactic;
005    
006    import org.apache.uima.jcas.JCas;
007    import org.apache.uima.jcas.JCasRegistry;
008    import org.apache.uima.jcas.cas.TOP;
009    import org.apache.uima.jcas.cas.TOP_Type;
010    
011    
012    /** This typed dependency structure was created to hold typed dependency information generated by the Stanford Parser.
013     * Updated by JCasGen Tue Aug 28 16:44:10 MDT 2007
014     * XML source: /home/williamb/eclipse/RELEASED_SOURCE_CODE/descriptors/typeSystem/CCPTypeSystem.xml
015     * @generated */
016    public class CCPTypedDependency extends TOP {
017      /** @generated
018       * @ordered 
019       */
020      public final static int typeIndexID = JCasRegistry.register(CCPTypedDependency.class);
021      /** @generated
022       * @ordered 
023       */
024      public final static int type = typeIndexID;
025      /** @generated  */
026      public              int getTypeIndexID() {return typeIndexID;}
027     
028      /** Never called.  Disable default constructor
029       * @generated */
030      protected CCPTypedDependency() {}
031        
032      /** Internal - constructor used by generator 
033       * @generated */
034      public CCPTypedDependency(int addr, TOP_Type type) {
035        super(addr, type);
036        readObject();
037      }
038      
039      /** @generated */
040      public CCPTypedDependency(JCas jcas) {
041        super(jcas);
042        readObject();   
043      } 
044    
045      /** <!-- begin-user-doc -->
046        * Write your own initialization here
047        * <!-- end-user-doc -->
048      @generated modifiable */
049      private void readObject() {}
050         
051     
052        
053      //*--------------*
054      //* Feature: governorTokenNum
055    
056      /** getter for governorTokenNum - gets 
057       * @generated */
058      public int getGovernorTokenNum() {
059        if (CCPTypedDependency_Type.featOkTst && ((CCPTypedDependency_Type)jcasType).casFeat_governorTokenNum == null)
060          jcasType.jcas.throwFeatMissing("governorTokenNum", "edu.uchsc.ccp.uima.annotation.syntactic.CCPTypedDependency");
061        return jcasType.ll_cas.ll_getIntValue(addr, ((CCPTypedDependency_Type)jcasType).casFeatCode_governorTokenNum);}
062        
063      /** setter for governorTokenNum - sets  
064       * @generated */
065      public void setGovernorTokenNum(int v) {
066        if (CCPTypedDependency_Type.featOkTst && ((CCPTypedDependency_Type)jcasType).casFeat_governorTokenNum == null)
067          jcasType.jcas.throwFeatMissing("governorTokenNum", "edu.uchsc.ccp.uima.annotation.syntactic.CCPTypedDependency");
068        jcasType.ll_cas.ll_setIntValue(addr, ((CCPTypedDependency_Type)jcasType).casFeatCode_governorTokenNum, v);}    
069       
070        
071      //*--------------*
072      //* Feature: dependentTokenNum
073    
074      /** getter for dependentTokenNum - gets 
075       * @generated */
076      public int getDependentTokenNum() {
077        if (CCPTypedDependency_Type.featOkTst && ((CCPTypedDependency_Type)jcasType).casFeat_dependentTokenNum == null)
078          jcasType.jcas.throwFeatMissing("dependentTokenNum", "edu.uchsc.ccp.uima.annotation.syntactic.CCPTypedDependency");
079        return jcasType.ll_cas.ll_getIntValue(addr, ((CCPTypedDependency_Type)jcasType).casFeatCode_dependentTokenNum);}
080        
081      /** setter for dependentTokenNum - sets  
082       * @generated */
083      public void setDependentTokenNum(int v) {
084        if (CCPTypedDependency_Type.featOkTst && ((CCPTypedDependency_Type)jcasType).casFeat_dependentTokenNum == null)
085          jcasType.jcas.throwFeatMissing("dependentTokenNum", "edu.uchsc.ccp.uima.annotation.syntactic.CCPTypedDependency");
086        jcasType.ll_cas.ll_setIntValue(addr, ((CCPTypedDependency_Type)jcasType).casFeatCode_dependentTokenNum, v);}    
087       
088        
089      //*--------------*
090      //* Feature: relation
091    
092      /** getter for relation - gets 
093       * @generated */
094      public String getRelation() {
095        if (CCPTypedDependency_Type.featOkTst && ((CCPTypedDependency_Type)jcasType).casFeat_relation == null)
096          jcasType.jcas.throwFeatMissing("relation", "edu.uchsc.ccp.uima.annotation.syntactic.CCPTypedDependency");
097        return jcasType.ll_cas.ll_getStringValue(addr, ((CCPTypedDependency_Type)jcasType).casFeatCode_relation);}
098        
099      /** setter for relation - sets  
100       * @generated */
101      public void setRelation(String v) {
102        if (CCPTypedDependency_Type.featOkTst && ((CCPTypedDependency_Type)jcasType).casFeat_relation == null)
103          jcasType.jcas.throwFeatMissing("relation", "edu.uchsc.ccp.uima.annotation.syntactic.CCPTypedDependency");
104        jcasType.ll_cas.ll_setStringValue(addr, ((CCPTypedDependency_Type)jcasType).casFeatCode_relation, v);}    
105      }
106    
107
```
